# Brazil HARMONY: Improved Satisfaction With Facial Appearance and Psychosocial and Social Well-Being Following Combined Facial Aesthetic Treatment

**DOI:** 10.1093/asjof/ojag159

**Published:** 2026-09-07

**Authors:** Ada Trindade de Almeida, Lilia Ramos dos Santos Guadanhim, Ediléia Bagatin, Vivian Barzi Loureiro, Fabiana Wanick, Bruna Felix Bravo, Raquel de Melo Carvalho, Leticia Oba Galvão, Régia Patriota, Graeme Kerson, Shirley Chung, Camila Cazerta de Paula Eduardo

## Abstract

**Background:**

Multimodal facial aesthetic treatment has potential benefits that extend beyond facial enhancements.

**Objectives:**

The aim of this study was to assess satisfaction with improvement in facial appearance and quantify the psychological, social, and emotional impact of comprehensive, multimodal, full-face aesthetic injectable treatment.

**Methods:**

In this prospective, open-label, multisite study, participants (*n* = 64) sequentially received a combination of a hybrid injectable containing hyaluronic acid (HA) plus calcium hydroxyapatite, HA injectables, and onabotulinumtoxinA over 5 months. The primary efficacy endpoint was change in FACE-Q Satisfaction with Facial Appearance Overall scale score. Secondary endpoints included the following assessments: FACE-Q Aging Appraisal, Psychological Function, Social Function, and Satisfaction with Skin; Global Aesthetic Improvement Scale (GAIS); Periorbital Aesthetic Appearance Questionnaire (PAAQ); and participant self-perception of facial age. Outcomes were measured as change in score from baseline to final visit.

**Results:**

Participants demonstrated significant improvement on the FACE-Q Satisfaction with Facial Appearance Overall scale (mean score increase = 46.6; *P* < .0001). In secondary efficacy analyses, significant improvement was observed on FACE-Q Psychological Function, Social Function, Aging Appraisal, and Satisfaction with Skin scales (*P* < .0001) and PAAQ (*P* < .0001). All investigators and participants noted improvement in facial appearance on the GAIS. A majority (85.5%) of participants reported improvement in age-related facial appearance. Most participants documented mild or moderate injection-site reactions (ISRs), and mean ISR durations ranged from 1.0 to 14.5 days.

**Conclusions:**

The Brazil HARMONY study provides further support that a combination of HA injectables, biostimulators, and neuromodulators leads to significant improvements in participant satisfaction and psychological and social well-being on validated patient-reported outcome scales.

**Level of Evidence: 4 (Therapeutic):**

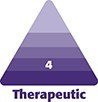

The most frequently cited reasons for seeking cosmetic procedures are a desire to “feel more confident,” “do something for myself,” “appear more attractive,” and “look as young as I feel or better for my age.”^1^ Studies have shown that self-perception of appearance is directly linked to self-esteem.^2,3^ In a meta-analysis, there was a positive correlation between self-rated attractiveness and self-esteem (*r* = 0.32), which was significantly stronger in women (*r* = 0.33) than men (*r* = 0.27).^3^ When an individual is dissatisfied with their appearance, it can negatively affect their mood, confidence, and self-worth. Opposingly, positive self-perception of appearance can result in increased self-confidence and self-esteem.

Several minimally invasive procedures are available to provide individuals with natural-appearing results for facial rejuvenation, thereby improving confidence and self-acceptance. OnabotulinumtoxinA (Botox Cosmetic; Allergan Aesthetics, an AbbVie company, Irvine, CA) is an effective, widely used treatment option for upper facial lines, including forehead lines, crow's feet, and glabellar lines.^4,5^ Hyaluronic acid (HA)-based injectables include VYC-12L (Juvéderm Volite/Skinvive) for superficial cutaneous depressions and skin quality improvement, VYC-15L (Juvéderm Volbella) for the treatment of fine lines and medium-sized skin depressions, VYC-17.5L (Juvéderm Volift) for deep skin depressions and face contouring and volume restoration, VYC-20L (Juvéderm Voluma) for volume restoration, and VYC-25L (Juvéderm Volux) to restore and generate facial volume in the chin and mandible areas (all products, Allergan Aesthetics, an AbbVie company). A safe and effective treatment option for facial soft tissue augmentation and skin architecture improvement is a hybrid injectable containing both HA for an immediate visible aesthetic effect and calcium hydroxyapatite microspheres for fibroblast stimulation (HA-CaHA), leading to the production of endogenous collagen and other extracellular matrix components (HArmonyCa; Allergan Aesthetics, an AbbVie company).^6-8^

A multimodal treatment approach that considers the whole face allows for a personalized, coordinated plan to achieve natural, balanced, and comprehensive facial rejuvenation and has been shown to increase satisfaction and psychosocial function and achieve a younger appearance.^9-12^ The US HARMONY study was a multicenter, single-blind trial that showed high participant satisfaction with facial appearance and significant psychosocial and social benefits after 4 months of treatment with onabotulinumtoxinA, HA injectables, and bimatoprost 0.03% ophthalmic solution for the treatment of facial rhytids, facial lines and folds and volume restoration, and eyelash hypotrichosis, respectively (all products, Allergan Aesthetics, an AbbVie company).^9^ The Canada HARMONY study, a single-arm multisite study of onabotulinumtoxinA, HA injectables, and deoxycholic acid injections (all products, Allergan Aesthetics, an AbbVie company), demonstrated that multimodal treatment to the face and submental areas led to significant improvements in satisfaction with facial appearance and psychological and social functions.^12^ Results from these studies highlight the significant improvements in physical appearance provided by minimally invasive multimodal facial treatment and the importance of appearance to an individual's psychosocial well-being.

Few studies have quantified the impact of treatment with multiple approved aesthetic products on psychosocial patient outcomes. The initial US HARMONY study assessed the overall aesthetic impact of treating individuals with several modalities to achieve the individual's desired treatment outcomes; however, it did not include the use of a biostimulator such as HA-CaHA, HA fillers were limited to midface volume loss, and onabotulinumtoxinA was limited to the treatment of glabellar lines and crow's feet.^9^ The Brazil HARMONY study expands upon the literature with the inclusion of additional products tested and face areas treated in a diverse population with a broader range of Fitzpatrick skin types and more men. The primary objective of this study was to quantify the psychological, social, and emotional impact of comprehensive, multimodal aesthetic treatment of the face and to assess the satisfaction of improvements in overall facial appearance.

## METHODS

### Study Design

This was a Phase 4, prospective, single-arm, open-label, postmarketing study conducted at 6 sites in Brazil from February 15, 2023 to May 10, 2024 (NCT04609020). Study recruitment strategies also targeted men, who are typically underrepresented in aesthetic studies. Participants received combined treatment (all products, Allergan Aesthetics, an AbbVie company) that included HA-CaHA with lidocaine, HA injectables with lidocaine (VYC-25L, VYC-20L, VYC-17.5L, VYC-15L, and VYC-12L), and onabotulinumtoxinA. Treatments were administered over a 5-month period, and outcomes were compared with baseline before treatment. The study protocol, informed consent form, and treating investigators were approved by Brazil's National Research Ethics Commission (CONEP) and institutional ethics committees. All participants provided written informed consent. This study was conducted in accordance with standard operating procedures that met the International Council for Harmonisation E6 Good Clinical Practice guidelines and the most recent version of the Declaration of Helsinki.

### Participants

Participants included in the study were aged 35 to 65 years at the time of screening and qualified for onabotulinumtoxinA treatments in at least 1 of the following approved areas: glabellar region (score of 2 [moderate] or 3 [severe] during maximum muscle contraction on the facial wrinkle scale [FWS]), crow's feet (score of 2 or 3 during maximum smile on the FWS), and forehead (score of 2 or 3 at maximum eyebrow elevation on the FWS). Participants were also qualified to receive at least 2 HA dermal injectable treatments in at least 2 different aesthetic areas. Participants agreed to refrain from any additional facial procedures or treatments and to avoid direct and prolonged sun exposure to the face, including tanning beds, at any time during the study period. Key exclusion criteria included a BMI >30 kg/m^2^, previous treatment with onabotulinumtoxinA or any other botulinum toxin product for any condition or mesotherapy or skin resurfacing within 6 months before study enrollment, or temporary or semipermanent facial or neck dermal filler treatment within 12 months before enrollment. Participants were also excluded if they had undergone or were planning to undergo surgery of the face and/or neck, tissue grafting, or tissue augmentation procedures.

### Timing of Treatments

**Figure 1.**
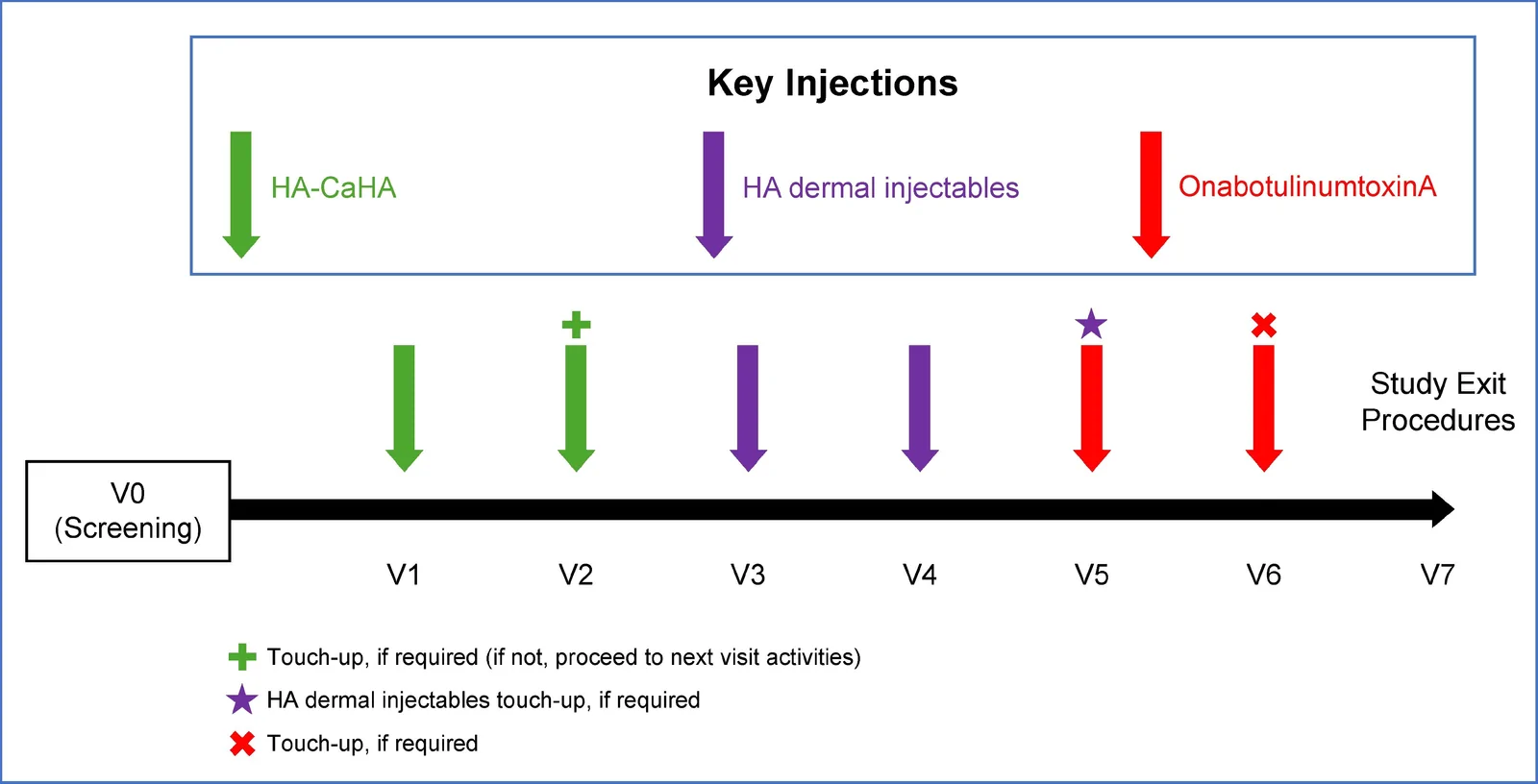
Study design. V0 may have been combined with V1 if judged by the investigator to be appropriate and acceptable for the participant. V2 to V5 occurred within 30 ± 7 days of the previous visit and V6 and V7 occurred within 15 ± 7 days. Treatment with HA-CaHA was optional and occurred on V1. V3 was the first treatment day for hyaluronic acid injectables, and V5 was the first treatment day for onobotulinumtoxinA. V7 was the final study visit when primary and secondary endpoints were collected. HA, hyaluronic acid; HA-CaHA, combined injectable containing hyaluronic acid plus calcium hydroxyapatite; V, visit.

The timing of study treatments is shown in Figure 1. All 7 visits were spaced ∼1 month apart, except for the final 2 study visits, which took place ∼15 days after the previous visit. Optional treatment with HA-CaHA occurred during Visit 1 with a subsequent touch-up at Visit 2. The volume of HA-CaHA injections was determined by the investigator and did not exceed 5 mL for the combined initial and touch-up treatments. Any participants not receiving the optional touch-ups or HA-CaHA injectables proceeded to the next treatment visit. HA dermal injectables were given on Visits 3 and 4, with a subsequent touch-up on Visit 5, if needed. The appropriate volume to be injected was determined by the investigator and did not exceed 4 mL for the combined initial visit and touch-up for VYC-12L, VYC-17.5L, VYC-20L, and VYC-25L, or 2 mL for VYC-15L. After treatment with HA-CaHA and HA injectables, participants received at least 1 treatment with onabotulinumtoxinA to the glabellar, crow's feet, or forehead regions, and, according to the investigator's evaluation during Visit 5, with an optional touch-up at Visit 6. Posttreatment efficacy assessments were given at the final study visit (Visit 7).

### Assessments

The primary efficacy endpoint was the change from baseline to the final study visit in the Rasch-transformed score of the validated FACE-Q Satisfaction with Facial Appearance Overall scale.^13^ This 10-item scale evaluated the concepts of facial symmetry, balance, proportion, freshness, rested look, profile, and appearance in photographs, when waking up, at the end of the day, and under bright lights. Each item was rated by the participant on a 4-point scale of 1 = very dissatisfied, 2 = somewhat dissatisfied, 3 = somewhat satisfied, and 4 = very satisfied. The raw scale summed scores, ranging from 10 to 40, were converted to a Rasch-transformed score ranging from 0 to 100, with higher scores indicating a better outcome.

Key secondary efficacy endpoints included the following 4-point FACE-Q scales: Aging Appraisal (7 items assessing patient feelings about how old their face looks in photos or in a mirror), Psychological Function (10 items assessing how patients feel about themselves when considering their facial appearance), Social Function (8 items assessing how a patient's facial appearance affects their confidence in social situations), and Satisfaction with Skin (12 items assessing the patient's satisfaction with the appearance and complexion of their facial skin). These endpoints were measured as the change from baseline in Rasch-transformed score to the final study visit. Improvements in the FACE-Q Psychological Function, Social Function, and Satisfaction with Skin scale scores were associated with positive responses (agreement/satisfaction), and improvement in the FACE-Q Aging Appraisal scale score was associated with negative responses (disagreement). Secondary endpoints also included investigator and participant assessment of improvement from baseline to the final study visit on the 5-point Global Aesthetic Improvement Scale (GAIS) measuring the level of improvement to the patient's facial lines, the change in participant satisfaction from baseline to the final study visit on the validated 5-point Periorbital Aesthetic Appearance Questionnaire (PAAQ; 9 questions assessing the patient's periorbital area over the past 7 days according to psychological, appearance, and coping domains), and participant assessment of age-related facial appearance as measured by self-perception of age recorded at baseline and the final study visit. In the assessment of age-related facial appearance, participants selected 1 of 3 options (“I look my current age,” “I look __ years younger,” and “I look __ years older”) based on their self-perception of facial appearance compared with their age.

### Safety

Safety endpoints included the number and percentage of participants who experienced any adverse events, injection-site reactions (ISRs), or device deficiencies, which were recorded daily in participant diaries. All adverse events, including ISRs, were collected from study enrollment through the final study visit.

### Statistical Analysis

Data were summarized with descriptive statistics (mean; standard deviation [SD]; standard error of the mean; median, minimum, and maximum for continuous variables; and *n* [%] for categorical variables). The full analysis population was used in the assessment of participant demographics and baseline characteristics and included all participants who enrolled in the study and had at least 1 posttreatment efficacy assessment at the final visit. The evaluable population was used for the efficacy analyses and included all participants in the full analysis set who received study products (HA injectables and onabotulinumtoxinA) and had no significant protocol violations or deviations that would have affected efficacy endpoints. The safety population used in the assessment of product safety included all participants who received any study product during the study period.

A planned sample size of 60 participants was determined based on a 1-sample, 1-sided *t* test at a 0.025 significance with assumptions based on FACE-Q scale results from a previous HARMONY study, with a potential dropout rate of 20%.^9^ Shapiro–Wilk test was used to check the normality assumption for the primary and secondary efficacy analyses. If the normality assumptions were met, a 1-sample, 1-sided *t* test at 0.025 significance was conducted. If the normality assumptions were not met, a Wilcoxon signed-rank test was performed. All CIs were 1-sided 97.5% CIs.

## RESULTS

### Participants

A total of 65 individuals were screened for study participation, and 64 participants were enrolled in the study. Two participants (3.1%) were subsequently excluded from efficacy analyses because of protocol violations. Demographic characteristics of study participants are shown in Table 1. The mean age was 48.4 years (SD = 7.4 years), the majority were female (79.7%), and there was a diverse representation of Fitzpatrick skin phototypes (II, 15.6%; III, 39.1%; IV, 26.6%; V, 15.6%; VI, 3.1%).

**Table 1.** Participant Demographics

Characteristic	Overall (*N* = 64)	Female (*n* = 51)	Male (*n* = 13)
Age, years			
Mean (SD)	48.4 (7.4)	48.9 (7.3)	46.3 (7.8)
Min, max	36, 62	36, 62	36, 59
Female, *n* (%)	51 (79.7)	—	—
BMI, kg/m^2^			
Mean (SD)	24.4 (2.5)	24.2 (2.5)	25.0 (2.6)
Min, max	19.1, 29.9	19.1, 29.9	19.7, 29.8
Fitzpatrick skin phototype, *n* (%)			
I	0	0	0
II	10 (15.6)	9 (17.6)	1 (7.7)
III	25 (39.1)	21 (41.2)	4 (30.8)
IV	17 (26.6)	14 (27.5)	3 (23.1)
V	10 (15.6)	7 (13.7)	3 (23.1)
VI	2 (3.1)	0	2 (15.4)

BMI, body mass index; SD, standard deviation.

### Study Treatments

**Figure 2.**
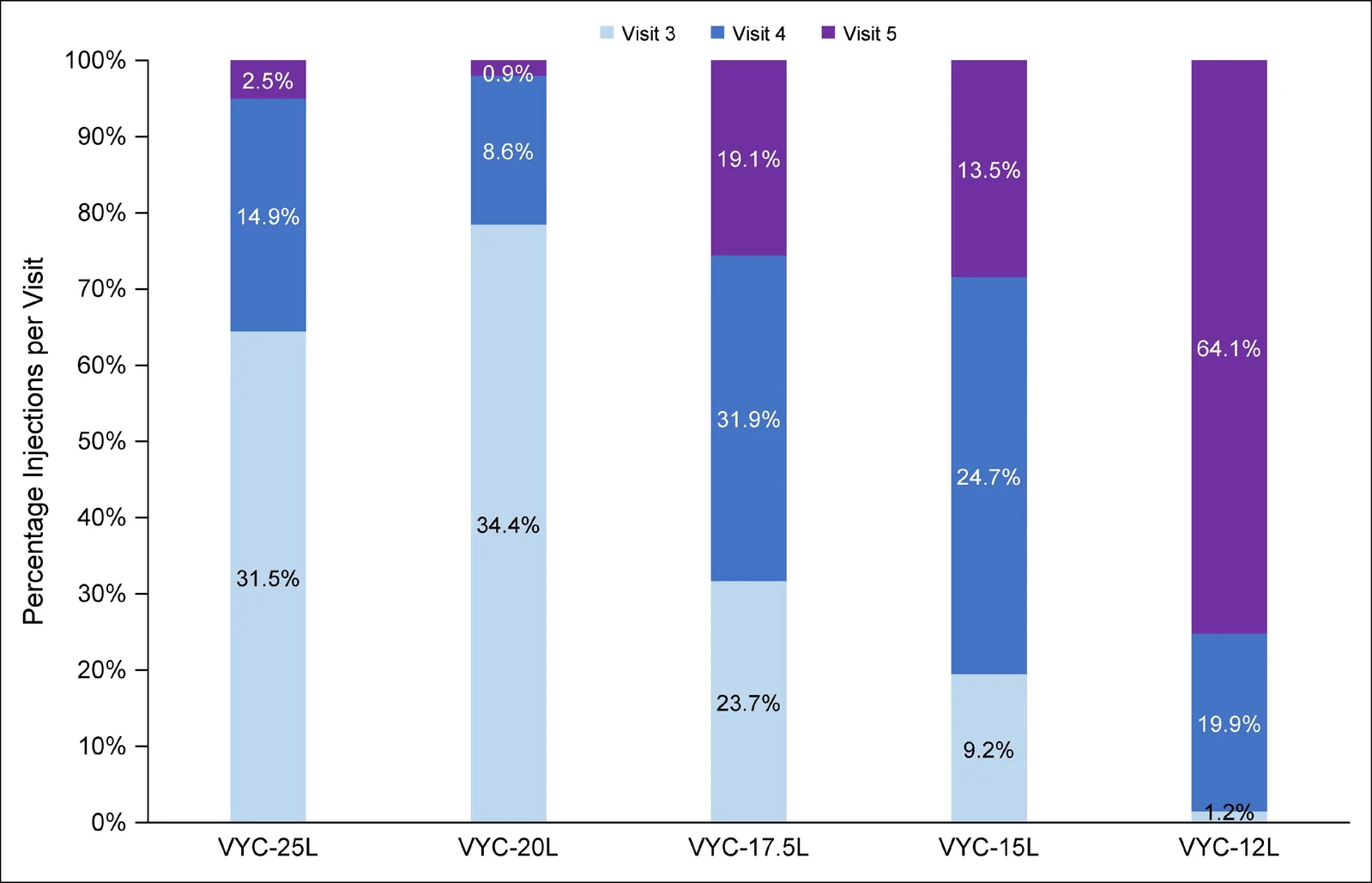
Proportion of injections per visit by hyaluronic acid injectable.

**Figure 3.**
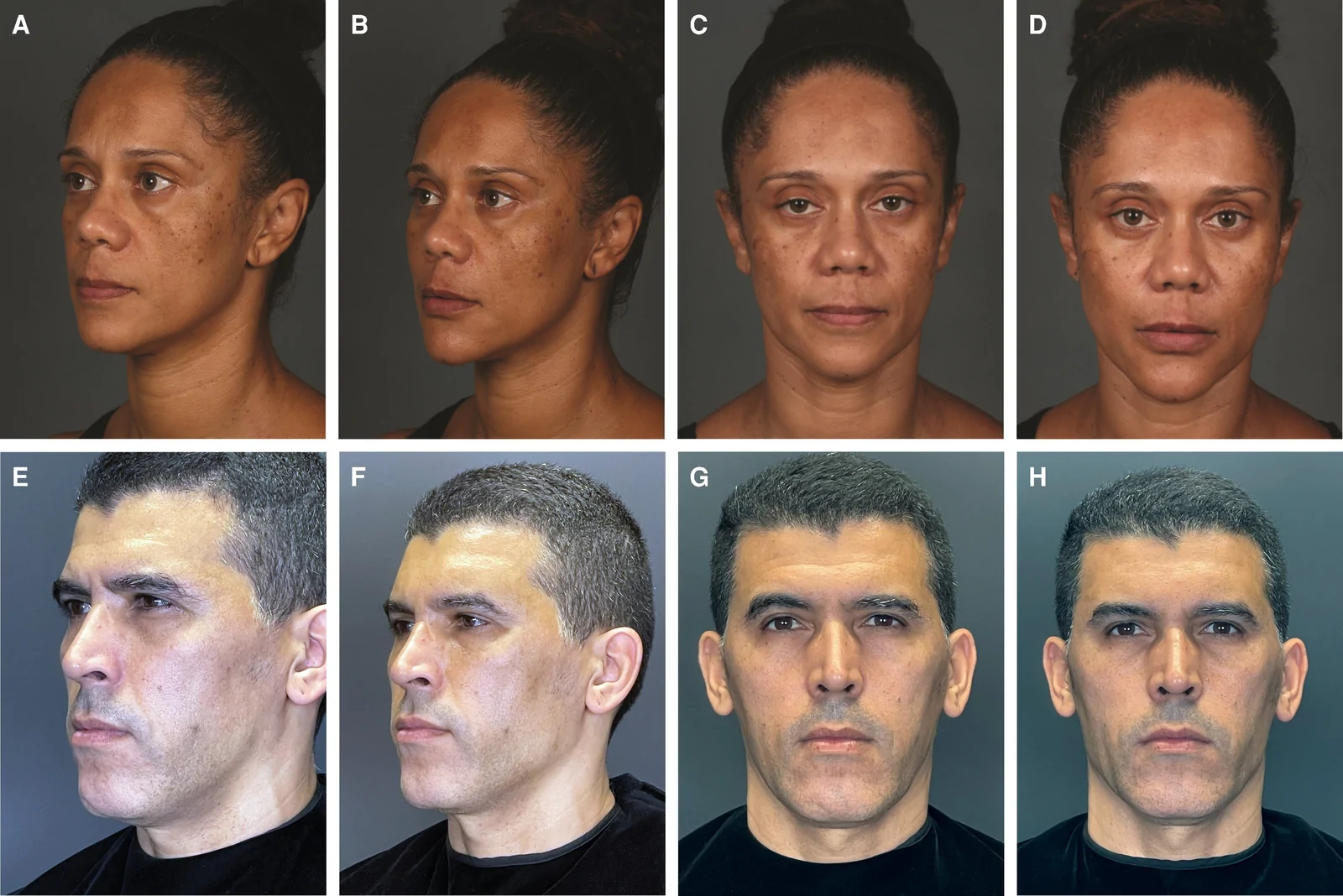

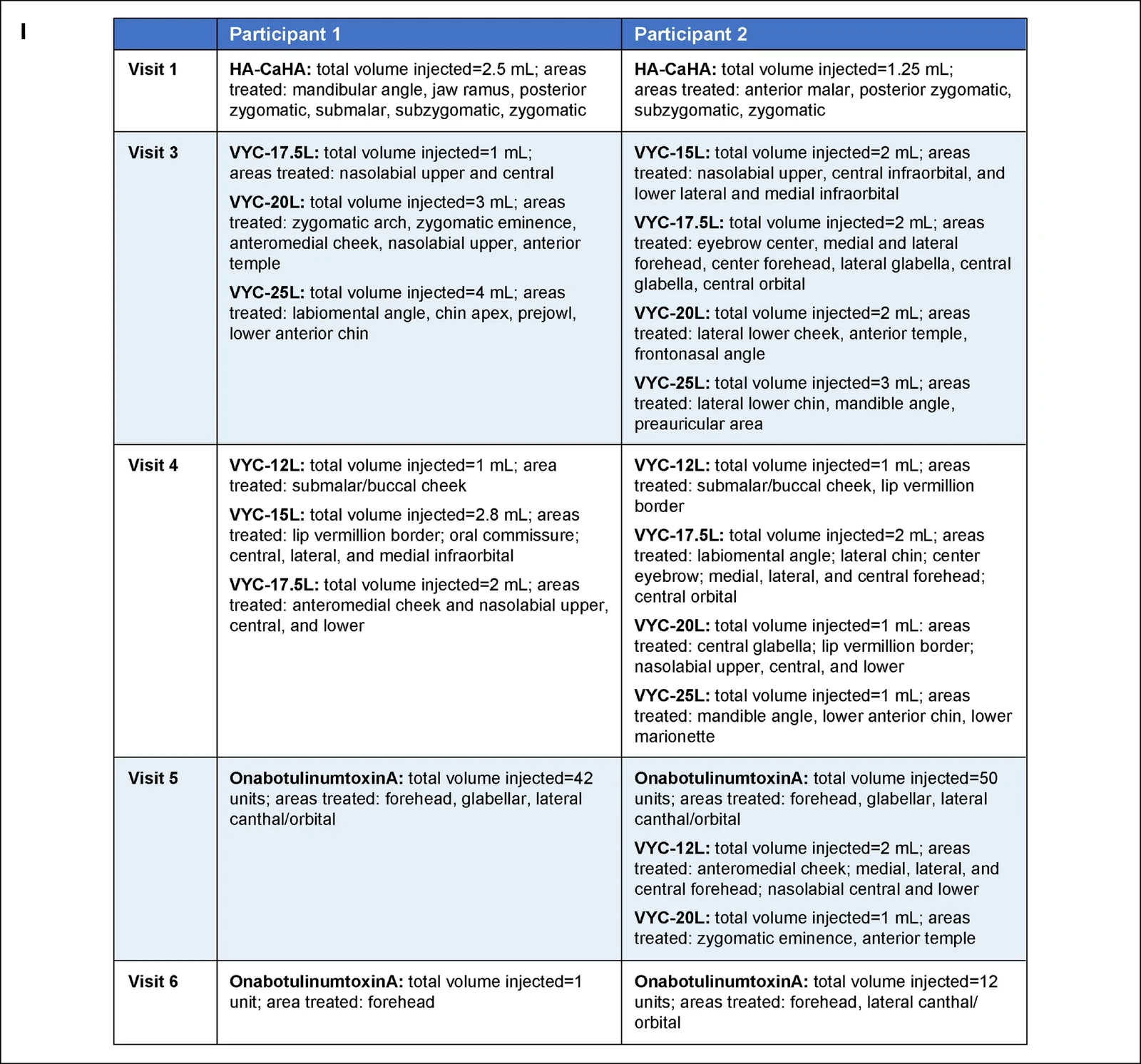
Participant images at baseline and follow-up. All sites were trained on image capture to standardize the photographs. Images that did not meet the standards of the study were retaken. Participant 1 was a 48-year-old female shown (A, C) at baseline and (B, D) at the final study visit 3 months after the first treatment session. Participant 2 was a 44-year-old male (E, G) at baseline and (F, H) at the final study visit 5 months after the first treatment session. (I) Corresponding regimens for the 3 participants.

Participants were treated on average for 151.5 days (SD = 24.6 days). Over the course of the study, all participants (*n* = 64) received HA-CaHA with a mean injected volume of 4.02 mL. Participants also received a mean combined injected volume of 15.0 mL for all HA injectables (3.5 mL VYC-25L [*n* = 63], 3.8 mL VYC-20L [*n* = 64], 3.6 mL VYC-17.5L [*n* = 64], 1.8 mL VYC-15L [*n* = 62], and 2.7 mL VYC-12L [*n* = 59]) and a mean combined injected volume of 75.7 U of onabotulinumtoxinA (25.0 U both sides lateral canthal, 29.0 U glabella, 22.1 U forehead). Participants tended to receive the structural HA injectables (ie, VYC-25L, VYC-20L, and VYC-17.5 L for volume restoration of the jawline, cheeks/chin, and smile lines, respectively) first, followed by those targeting the refinement of fine lines/wrinkles and skin quality (ie, VYC-15L and VYC-12L), as shown in Figure 2. Select participant images and corresponding treatments are shown in Figure 3.

### Primary Endpoint Assessment

**Figure 4.**
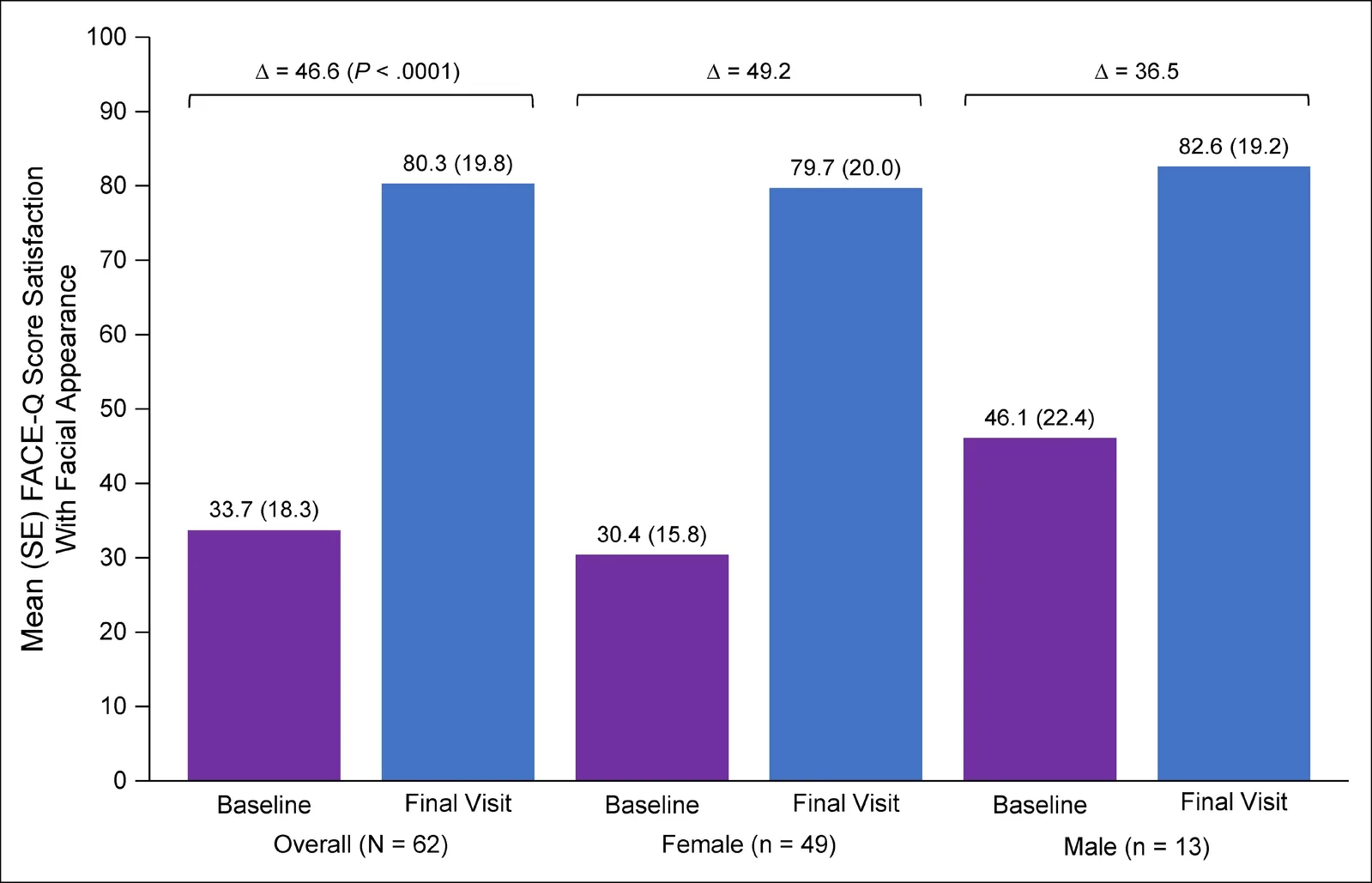
Mean (standard error [SE]) FACE-Q Satisfaction with Facial Appearance scale overall score at baseline and final visit. Statistical testing was not conducted on the differences between sex subgroups.

Participants demonstrated significant (*P* < .0001) improvement in the FACE-Q Satisfaction with Facial Appearance Overall scale score from baseline to the final study visit, with a mean increase of 46.6 (SD = 23.6) on a scale of 100 (Figure 4). On average, the majority of participant responses to each question changed from “very dissatisfied” and “somewhat dissatisfied” at baseline to “very satisfied” at the final study visit, indicating improvement in the participants' perception of overall facial appearance (Supplemental Table 1). Baseline scores were 30.4 (SD = 15.8) for females and 46.1 (SD = 22.4) for males, increasing to 79.7 (SD = 20.0) and 82.6 (SD = 19.2) among females and males, respectively, at the final study visit (Figure 4).

### Secondary Endpoint Assessments

#### FACE-Q Scale Scores

**Figure 5.**
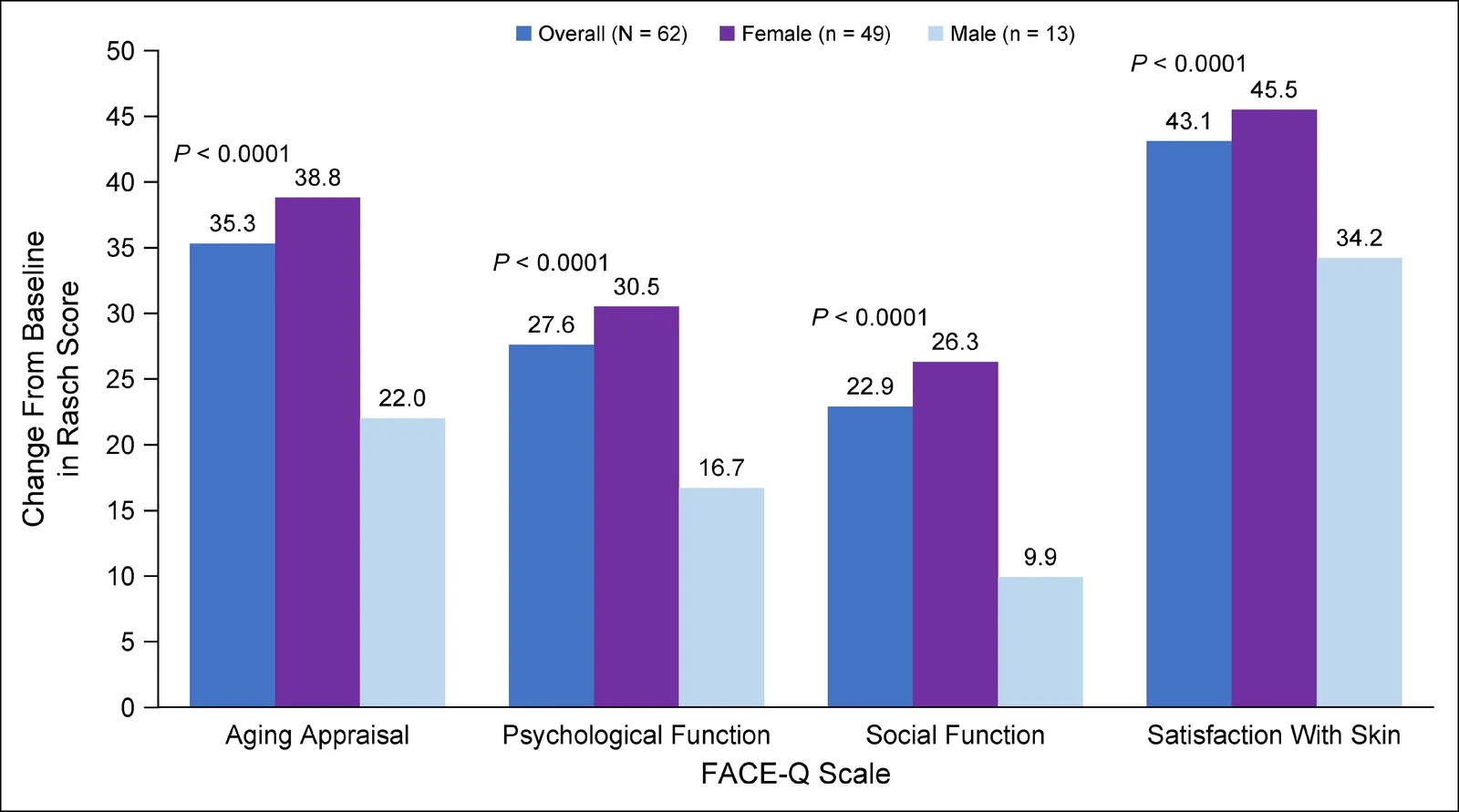
Mean change from baseline in FACE-Q Aging Appraisal, Psychological Function, Social Function, and Satisfaction with Skin scales after multimodal facial aesthetic treatment. *P* values were calculated based on a 1-sided *t* test or Wilcoxon signed-rank test at the 0.025 significance level for the overall change in score.

Significant improvements on the Aging Appraisal, Psychological Function, Social Function, and Satisfaction with Skin scales were demonstrated after multimodal treatment (Figure 5). Mean changes from baseline to the final study visit showed significant improvement on the Aging Appraisal scale (*P* < .0001), with a mean increase in score of 35.3 (SD = 22.4) on a scale of 100. Males had a numerically higher mean baseline score than females (65.4 [SD = 27.5] vs 45.2 [SD = 19.4]), but a numerically lower mean score increase after the final study visit (22.0 [SD = 26.5] vs 38.8 [20.0]). On average, most participant responses to each question on the Aging Appraisal scale were “definitely disagree” at the final study visit, indicating improvement or a more favorable self-perception of how old the participant appeared (Supplemental Table 2).

On the Psychological Function scale, the mean increase in score from baseline to the final study visit was 27.6 (SD = 23.2, *P* < .0001) on a scale of 100. Again, males had a numerically higher mean baseline score than females (76.0 [SD = 23.4] vs 56.9 [SD = 21.3]), but a lower mean score increase following the final study visit. Males had a mean increase of 16.7 (SD = 23.5) and females had an increase of 30.5 (SD = 22.5). On average, most participant responses to each question on the Psychological Function scale were “definitely agree” at the final study visit, indicating improvement in psychological well-being as a result of changes in their appearance (Supplemental Table 3).

On the Social Function scale, the mean score increase was 22.9 (SD = 20.6, *P* < .0001) on a scale of 100 from baseline to the final study visit. Males had a numerically higher mean baseline score than females (73.2 [SD = 23.5] vs 55.5 [SD = 17.5]), but a lower mean increase after the final study visit (9.9 [SD = 16.2] vs 26.3 [SD = 20.3], respectively). On average, the majority of participant responses to most questions on the Social Function scale ranged from “somewhat disagree” or “somewhat agree” at baseline to “definitely agree” at the final study visit, indicating improvement in social interactions as a result of changes in their appearance (Supplemental Table 4).

On the Satisfaction with Skin scale, the mean score increase from baseline to the final study visit was 43.1 (SD = 22.3, *P* < .0001) on a scale of 100. Males had a numerically higher mean baseline score than females (48.5 [SD = 18.2] vs 31.2 [SD = 16.7]), but a numerically lower mean score increase following the final study visit. Males had a mean increase of 34.2 (SD = 22.5) and females had an increase of 45.5 (SD = 21.8). On average, most participant responses to most questions on the Satisfaction with Skin scale ranged from “very dissatisfied” or “somewhat dissatisfied” at baseline to “very satisfied” at the final study visit, indicating improvement in satisfaction with the appearance of their facial skin (Supplemental Table 5).

#### Global Aesthetic Improvement Scale

The investigator-assessed GAIS reported improvement in the facial appearance of all participants from baseline to the final study visit (24.2% “improved” and 75.8% “much improved”). Similarly, all participants reported improvement in their facial appearance from baseline to the final study visit on the GAIS (17.7% “improved” and 82.3% “much improved”).

#### Periorbital Aesthetic Appearance Questionnaire

**Figure 6.**
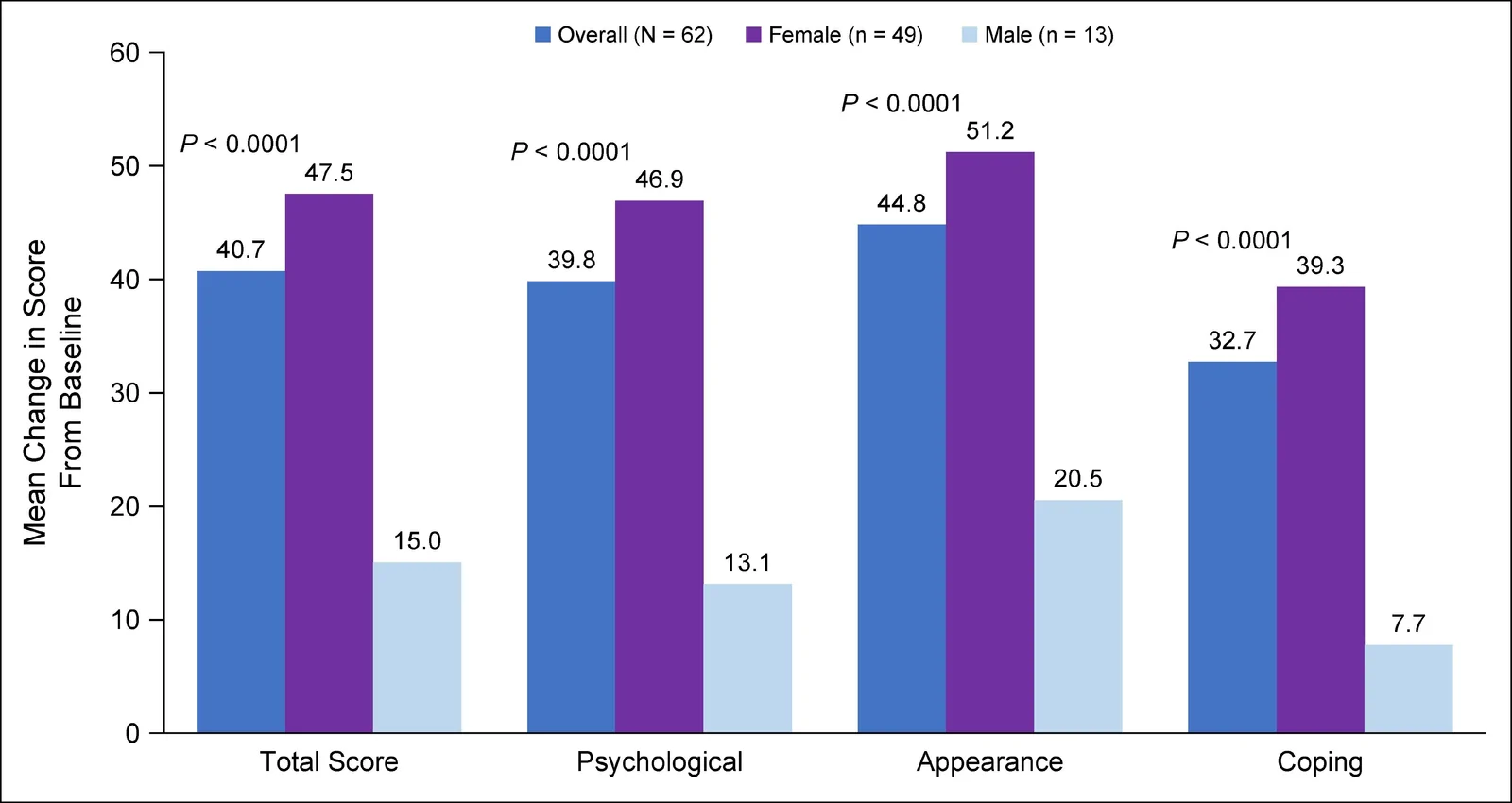
Mean change from baseline on the Periorbital Aesthetic Appearance Questionnaire after multimodal facial aesthetic treatment. *P* values were calculated based on a 1-sided *t* test or Wilcoxon singed-rank test at the 0.025 significance level for the overall change in score.

The mean transformed PAAQ score improved significantly from baseline to the final study visit overall (mean [SD] score change, −40.7 [24.5]; *P* < .0001) and in all 3 domains (psychological mean score change, −39.8 [25.0]; *P* < .0001; appearance mean score change, −44.8 [27.1]; *P* < .0001; and coping mean score change, −32.7 [29.9], *P* < .0001; Figure 6).

#### Age-Related Facial Appearance

**Figure 7.**
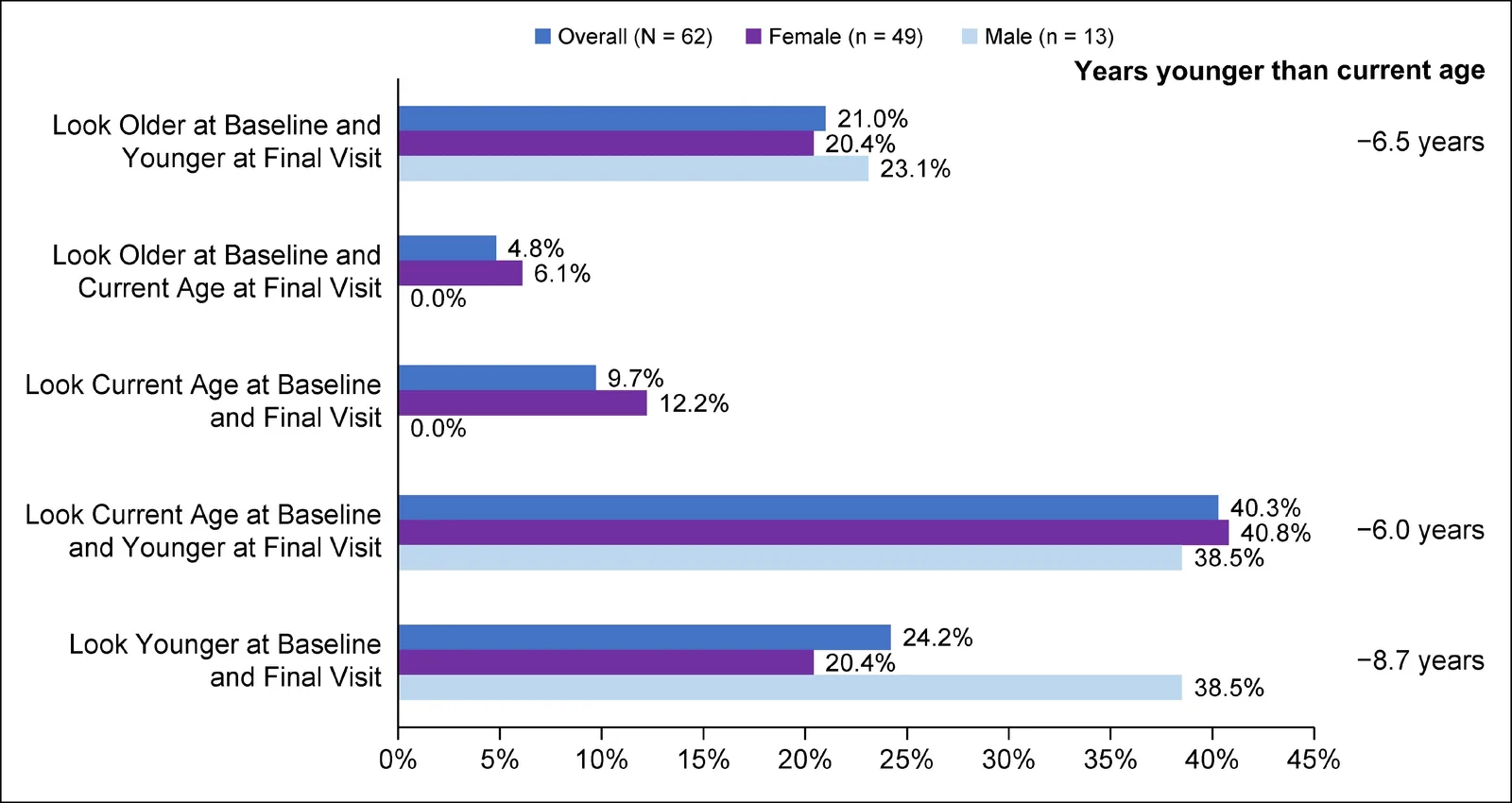
Self-perception of age. After multimodal aesthetic treatment, on average, participants rated themselves as 8.7 years (absolute difference, 3.4 years), 6.0 years, and 6.5 years (absolute difference, 12.6 years) younger in appearance among those who thought they looked younger at baseline, their current age at baseline, and older at baseline than their actual age, respectively. Those who looked older at baseline and their current age after treatment reported an absolute difference of 4.7 years younger than their baseline appearance.

A total of 85.5% of participants noted an improvement in their assessment of age-related facial appearance after multimodal aesthetic treatment (Figure 7). At baseline, 50.0% of participants responded that they looked their age, 24.2% that they looked younger than their age, and 25.8% that they looked older than their age. Comparatively, at the final study visit, 14.5% of participants responded that they looked their age, 85.5% that they looked younger than their age, and no participants reported that they looked older than their age. Participants reported looking 6.5 and 8.7 years younger, on average, among those who reported looking older at baseline and younger at the final study visit and those who reported looking younger at both baseline and final study visits, respectively.

### Safety

A total of 10 (15.6%) participants experienced a treatment-emergent adverse event (TEAE). There were no TEAEs that led to study discontinuation, TEAEs related to device malfunctions/deficiencies, or treatment-related serious TEAEs. Most TEAEs resolved in 4 to 7 days. Treatment-related TEAEs occurred in 3 (4.7%) participants because of HA injectables/HA-CaHA products. One case of edema of moderate severity occurred in a participant who received VYC-25L and HA-CaHA and lasted 4 days. One participant reported nodules in treated areas of mild severity after receiving HA-CaHA, VYC-12L, VYC-25L, VYC-17.5L, and VYC-20L, which resolved within 4 days. Lastly, 1 participant reported induration of moderate severity that lasted 12 days, lip edema of moderate severity that lasted 14 days, periorbital edema of moderate severity that lasted 6 days, and chin edema at the injection site of moderate severity that lasted 14 days after receiving VYC-12L, VYC-25L, VYC-17.5L, VYC-20L, and VYC-25L.

Nearly all participants reported at least 1 ISR, which was mostly mild or moderate in severity. The most common ISRs were tenderness (63.8%), pain (53.2%), swelling (40.4%), lumps (36.2%), and redness (17.0%). The mean time to symptom resolution ranged from 1.0 day (firmness) to 14.5 days (itching).

## DISCUSSION

Brazil HARMONY was a Phase 4, prospective, interventional, multicenter, postmarketing study that evaluated the psychological, emotional, and social impacts of multimodal aesthetic treatment of the face with HA-CaHA, HA injectables, and onabotulinumtoxinA. This study demonstrated that both men and women who received multimodal aesthetic facial treatment noted significant improvement in the satisfaction of their facial appearance on the FACE-Q Satisfaction with Facial Appearance scale. Participants also noted significant improvements on secondary efficacy measures, including the FACE-Q Aging Appraisal, Psychological Function, Social Function, and Satisfaction with Skin scales and the PAAQ validated patient-reported outcome scale. All participants and investigators noted improvement in facial appearance on the GAIS after treatment, with participants documenting slightly higher perceptions of improvement than investigators.

As aging of the face can adversely affect communication, attractiveness, and self-esteem, aesthetic treatment goals are not to look younger but rather to enhance and improve the appearance of oneself.^14^ The aging face can affect the perception of emotions and personality traits of an individual by others.^14,15^ For example, drooping of the mouth can create a frown, which may be misinterpreted as sadness or displeasure. Forehead lines and creases in the glabellar region can come across as anger or dissatisfaction. The sagging of the brow and eyelids may appear as exhaustion or drowsiness.^14,16^ These misinterpretations can affect the ability to communicate with others, thus creating social disconnect, anxiety, low self-esteem, and ultimately social isolation.^14^ In a meta-analysis, low self-esteem levels had consequential effects on social relationships, including those with the general population, parents, and peers.^17^

Individuals often seek aesthetic treatment to improve psychological and social well-being. In a multicenter, prospective observational study, participants noted psychological motivating factors in seeking minimally invasive cosmetic procedures, such as a desire to feel more confident (69.5%) and to feel happier or better overall or to improve quality of life (67.2%). Participants were also motivated to undergo cosmetic procedures for social reasons, such as a desire to look good when running into people they knew (56.6%), to feel less self-conscious (50.3%), to look good for social events (42.2%), and to make a better first impression (38.4%).^18^

In the current study, receipt of multimodal facial aesthetic treatment improved psychological well-being. On the FACE-Q Psychological Function scale, participants were more likely to feel attractive, confident, and like themselves after treatment. The proportion of participants who responded as “definitely agree” to the item “happy” increased from 38.7% at baseline to 83.9% at the final study visit. In regard to the item “accepting,” the proportion of participants who responded “definitely agree” increased from 21.0% at baseline to 74.2% at the final study visit. Additionally, the proportion of participants who responded “definitely agree” to the item “confident” increased from 33.9% at baseline to 80.6% at the final study visit. On the PAAQ scale, participants noted less self-consciousness and sadness, a decreased need to wear makeup, and a decreased desire to do something to take attention away from the eye area after treatment. Similar findings have been reported in previous HARMONY studies. In the US HARMONY study, participants experienced a 19.9-point increase in scores on the FACE-Q Psychological Well-being scale from baseline to the final study visit, with responses moving from “somewhat agree” to “definitely agree” on 8 of 10 questions after multimodal facial aesthetic treatment.^10^ In the Canada HARMONY study, participants had a mean score increase of 18.8 points on the FACE-Q Psychological Function scale. In 8 of 10 questions that assessed participants' positive feelings about themselves, responses changed from “somewhat agree” to “definitely agree” after multimodal facial aesthetic treatment.^12^

Receipt of multimodal treatment also improved social well-being in this study. On the FACE-Q Social Function Scale, more participants reported feeling comfortable walking into a room full of strangers and meeting new people and feeling confident in new social situations. The number of participants who responded “definitely agree” to the item “make friends” increased from 35.5% at baseline to 66.1% at the final study visit. Additionally, the percentage of participants who responded “definitely agree” to the item “first impressions” increased from 14.5% at baseline to 56.5% at the final study visit. Similarly, the percentage of participants who responded “definitely agree” to the item “new person” increased from 16.1% to 61.3% at baseline to the final study visit, respectively. Previous studies have also demonstrated improvements in social well-being after facial aesthetic treatment. In the US HARMONY study, the overall mean scores on the FACE-Q Social Confidence scale improved by 18.2 points from baseline to the final visit. On 6 of the 8 questions, responses improved from “somewhat agree” at baseline to “definitely agree” after multimodal facial aesthetic treatment.^10^ Similarly, in the Canada HARMONY study, patients experienced a 16.1-point increase in scores from baseline to the final visit on the FACE-Q Social Function scale. Participants noted an improvement from “somewhat agree” to “definitely agree” on 5 of 8 questions after multimodal facial aesthetic treatment.^12^ Dayan et al used pretreatment and posttreatment images from the US HARMONY study and blinded respondents to evaluate the impact of multimodal aesthetic treatment on the perception of an individual in different social contexts. Respondents noted that individuals in the posttreatment images appeared to possess more positive character traits; were more socially adept, younger, attractive, and successful at attracting others; had a higher income; and were more educated than individuals in the pretreatment images.^15^

In this study, both men and women experienced significant benefits to their appearance, psychological well-being, and social well-being after multimodal aesthetic treatment; however, the treatment effects (ie, mean changes from baseline) were lower among males than females. One explanation for this is that males reported higher baseline satisfaction with their facial appearance compared with females. This is in line with other studies that have shown that men tend to have higher opinions of their attractiveness than women.^19,20^ Additionally, women tend to have lower self-esteem than men, and self-esteem in women is more closely related to perceived physical attractiveness.^19^ This may explain why women reported much higher improvements than men in psychological and social well-being following improvements in their facial appearance.

The results of the current study extend and support those from previous HARMONY studies to highlight how a comprehensive, multimodal treatment approach can improve appearance and positively affect the psychological and social well-being of the participant. The flexibility of dosing delivered over 4 or more sessions in this study showed that a multimodal treatment approach was well tolerated. The safety profile observed was similar to that of the individual products. The severity and duration of ISRs were similar to those reported in the US HARMONY and Canada HARMONY studies.^9,12^

This study had a few limitations. First, this was a nonrandomized study with no comparator treatment arm. Second, despite targeting men during enrollment, ∼80% of the study participants were women, limiting generalizability; however, this number is representative of the proportion of males who undergo nonsurgical aesthetic procedures.^21^ Third, investigator assessments and related outcomes were limited to the GAIS. This study relied mostly on self-reporting instruments, which may be biased given the subjective nature of the validated questionnaires and that patients were not blinded to treatment. Self-perceptions of one's appearance before and after treatment can vary by gender, ethnicity, treatment expectations, and cultural standards of beauty. Fourth, a multimodal treatment approach could also include energy-based devices with injectables, which would need further research into optimal sequencing and device selection. The treatments and sequence used within this study may not be an option for all patients because of factors such as cost or time, but the overall approach for treatment planning is important for any aesthetic patient.

## CONCLUSIONS

The Brazil HARMONY study extends the US HARMONY and Canada HARMONY studies and provides further evidence that multimodal, minimally invasive aesthetic injectable treatments result in significant improvements in participant satisfaction with their facial appearance, psychological and social well-being, and self-perception of appearance in the context of facial aging on validated patient-reported outcome scales. Treatments were well tolerated, and most adverse events were mild in severity.

## Supplementary Material

ojag159_Supplementary_Data

## Data Availability

AbbVie is committed to responsible data sharing regarding the clinical trials we sponsor. This includes access to anonymized, individual, and trial-level data (analysis data sets), as well as other information (eg, protocols, clinical study reports, synopses, or statistical analysis plans), as long as the trials are not part of an ongoing or planned regulatory submission. These clinical trial data can be requested by any qualified researchers who engage in rigorous, independent, scientific research, and will be provided following review and approval of a research proposal, Statistical Analysis Plan (SAP), and execution of a Data Use Agreement (DUA). Data requests can be submitted at any time after approval in the US and Europe and after acceptance of this manuscript for publication. The data will be accessible for 12 months, with possible extensions considered. For more information on the process or to submit a request, visit the following link: https://vivli.org/ourmember/abbvie/ then select “Home.”
