# Supplementary material for: Brazil HARMONY: Improved Satisfaction With Facial Appearance and Psychosocial and Social Well-Being Following Combined Facial Aesthetic Treatment
Source: Aesthet Surg J Open Forum. 2026 Sep 7;8:ojag159. doi: 10.1093/asjof/ojag159 (PMC13603039; doi:10.1093/asjof/ojag159)
Supplement: Supplementary file 1 [file ojag159_supplementary_data.zip › ASJOF-26-0027_Brazil HARMONY_Supplementary Table 1.docx]

**Supplementary Table 1. FACE-Q Satisfaction with Facial Appearance Summary (n=62)**

|  | **Baseline, n (%)** | **Final Study Visit, n (%)** |
| --- | --- | --- |
| **FACE: symmetric** |  |  |
| Very dissatisfied | 14 (22.6) | 1 (1.6) |
| Somewhat dissatisfied | 25 (40.3) | 2 (3.2) |
| Somewhat satisfied | 21 (33.9) | 17 (27.4) |
| Very satisfied | 2 (3.2) | 42 (67.7) |
| **FACE: balanced** |  |  |
| Very dissatisfied | 11 (17.7) | 1 (1.6) |
| Somewhat dissatisfied | 25 (40.3) | 0 |
| Somewhat satisfied | 24 (38.7) | 17 (27.4) |
| Very satisfied | 2 (3.2) | 44 (71.0) |
| **FACE: proportioned** |  |  |
| Very dissatisfied | 13 (21.0) | 1 (1.6) |
| Somewhat dissatisfied | 24 (38.7) | 0 |
| Somewhat satisfied | 22 (35.5) | 18 (29.0) |
| Very satisfied | 3 (4.8) | 43 (69.4) |
| **FACE: end of day** |  |  |
| Very dissatisfied | 24 (38.7) | 1 (1.6) |
| Somewhat dissatisfied | 27 (43.5) | 2 (3.2) |
| Somewhat satisfied | 10 (16.1) | 29 (46.8) |
| Very satisfied | 1 (1.6) | 30 (48.4) |
| **FACE: fresh** |  |  |
| Very dissatisfied | 28 (45.2) | 1 (1.6) |
| Somewhat dissatisfied | 24 (38.7) | 1 (1.6) |
| Somewhat satisfied | 9 (14.5) | 18 (29.0) |
| Very satisfied | 1 (1.6) | 42 (67.7) |
| **FACE: rested** |  |  |
| Very dissatisfied | 31 (50.0) | 1 (1.6) |
| Somewhat dissatisfied | 18 (29.0) | 6 (9.7) |
| Somewhat satisfied | 12 (19.4) | 19 (30.6) |
| Very satisfied | 1 (1.6) | 36 (58.1) |
| **FACE: profile** |  |  |
| Very dissatisfied | 25 (40.3) | 1 (1.6) |
| Somewhat dissatisfied | 23 (37.1) | 5 (8.1) |
| Somewhat satisfied | 10 (16.1) | 13 (21.0) |
| Very satisfied | 4 (6.5) | 43 (69.4) |
| **FACE: photos** |  |  |
| Very dissatisfied | 26 (41.9) | 1 (1.6) |
| Somewhat dissatisfied | 26 (41.9) | 5 (8.1) |
| Somewhat satisfied | 9 (14.5) | 17 (27.4) |
| Very satisfied | 1 (1.6) | 39 (62.9) |
| **FACE: wake up** |  |  |
| Very dissatisfied | 30 (48.4) | 2 (3.2) |
| Somewhat dissatisfied | 21 (33.9) | 8 (12.9) |
| Somewhat satisfied | 10 (16.1) | 22 (35.5) |
| Very satisfied | 1 (1.6) | 30 (48.4) |
| **FACE: bright lights** |  |  |
| Very dissatisfied | 27 (43.5) | 1 (1.6) |
| Somewhat dissatisfied | 23 (37.1) | 6 (9.7) |
| Somewhat satisfied | 10 (16.1) | 17 (27.4) |
| Very satisfied | 2 (3.2) | 38 (61.3) |
