# Supplementary material for: Brazil HARMONY: Improved Satisfaction With Facial Appearance and Psychosocial and Social Well-Being Following Combined Facial Aesthetic Treatment
Source: Aesthet Surg J Open Forum. 2026 Sep 7;8:ojag159. doi: 10.1093/asjof/ojag159 (PMC13603039; doi:10.1093/asjof/ojag159)
Supplement: Supplementary file 1 [file ojag159_supplementary_data.zip › ASJOF-26-0027_Brazil HARMONY_Supplementary Table 2.docx]

**Supplementary Table 2. FACE-Q Aging Appraisal Summary (n=62)**

|  | **Baseline, n (%)** | **Final Study Visit, n (%)** |
| --- | --- | --- |
| **AGING APPRAISAL: don’t recognize** |  |  |
| Definitely agree | 1 (1.6) | 0 |
| Somewhat agree | 13 (21.0) | 0 |
| Somewhat disagree | 10 (16.1) | 3 (4.8) |
| Definitely disagree | 38 (61.3) | 59 (95.2) |
| **AGING APPRAISAL: look in mirror** |  |  |
| Definitely agree | 3 (4.8) | 0 |
| Somewhat agree | 15 (24.2) | 1 (1.6) |
| Somewhat disagree | 15 (24.2) | 10 (16.1) |
| Definitely disagree | 29 (46.8) | 51 (82.3) |
| **AGING APPRAISAL: bothered** |  |  |
| Definitely agree | 15 (24.2) | 0 |
| Somewhat agree | 21 (33.9) | 3 (4.8) |
| Somewhat disagree | 15 (24.2) | 10 (16.1) |
| Definitely disagree | 11 (17.7) | 49 (79.0) |
| **AGING APPRAISAL: older** |  |  |
| Definitely agree | 20 (32.3) | 0 |
| Somewhat agree | 18 (29.0) | 3 (4.8) |
| Somewhat disagree | 8 (12.9) | 10 (16.1) |
| Definitely disagree | 16 (25.8) | 49 (79.0) |
| **AGING APPRAISAL: worried** |  |  |
| Definitely agree | 24 (38.7) | 2 (3.2) |
| Somewhat agree | 25 (40.3) | 11 (17.7) |
| Somewhat disagree | 6 (9.7) | 13 (21.0) |
| Definitely disagree | 7 (11.3) | 36 (58.1) |
| **AGING APPRAISAL: photos** |  |  |
| Definitely agree | 26 (41.9) | 2 (3.2) |
| Somewhat agree | 25 (40.3) | 3 (4.8) |
| Somewhat disagree | 2 (3.2) | 12 (19.4) |
| Definitely disagree | 9 (14.5) | 45 (72.6) |
| **AGING APPRAISAL: reflection** |  |  |
| Definitely agree | 21 (33.9) | 1 (1.6) |
| Somewhat agree | 27 (43.5) | 8 (12.9) |
| Somewhat disagree | 5 (8.1) | 15 (24.2) |
| Definitely disagree | 9 (14.5) | 38 (61.3) |
