# Supplementary material for: Brazil HARMONY: Improved Satisfaction With Facial Appearance and Psychosocial and Social Well-Being Following Combined Facial Aesthetic Treatment
Source: Aesthet Surg J Open Forum. 2026 Sep 7;8:ojag159. doi: 10.1093/asjof/ojag159 (PMC13603039; doi:10.1093/asjof/ojag159)
Supplement: Supplementary file 1 [file ojag159_supplementary_data.zip › ASJOF-26-0027_Brazil HARMONY_Supplementary Table 3.docx]

**Supplementary Table 3. FACE-Q Psychological Function Summary (n=62)**

|  | **Baseline, n (%)** | **Final Study Visit, n (%)** |
| --- | --- | --- |
| **PSYCH: like** |  |  |
| Definitely disagree | 1 (1.6) | 0 |
| Somewhat disagree | 13 (21.0) | 0 |
| Somewhat agree | 17 (27.4) | 10 (16.1) |
| Definitely agree | 31 (50.0) | 52 (83.9) |
| **PSYCH: positive** |  |  |
| Definitely disagree | 1 (1.6) | 0 |
| Somewhat disagree | 12 (19.4) | 1 (1.6) |
| Somewhat agree | 24 (38.7) | 12 (19.4) |
| Definitely agree | 25 (40.3) | 49 (79.0) |
| **PSYCH: okay** |  |  |
| Definitely disagree | 4 (6.5) | 0 |
| Somewhat disagree | 14 (22.6) | 1 (1.6) |
| Somewhat agree | 24 (38.7) | 14 (22.6) |
| Definitely agree | 20 (32.3) | 47 (75.8) |
| **PSYCH: happy** |  |  |
| Definitely disagree | 0 | 0 |
| Somewhat disagree | 13 (21.0) | 1 (1.6) |
| Somewhat agree | 25 (40.3) | 9 (14.5) |
| Definitely agree | 24 (38.7) | 52 (83.9) |
| **PSYCH: comfortable** |  |  |
| Definitely disagree | 4 (6.5) | 0 |
| Somewhat disagree | 23 (37.1) | 1 (1.6) |
| Somewhat agree | 20 (32.3) | 12 (19.4) |
| Definitely agree | 15 (24.2) | 49 (79.0) |
| **PSYCH: accepting** |  |  |
| Definitely disagree | 8 (12.9) | 0 |
| Somewhat disagree | 21 (33.9) | 2 (3.2) |
| Somewhat agree | 20 (32.3) | 14 (22.6) |
| Definitely agree | 13 (21.0) | 46 (74.2) |
| **PSYCH: good** |  |  |
| Definitely disagree | 4 (6.5) | 0 |
| Somewhat disagree | 18 (29.0) | 1 (1.6) |
| Somewhat agree | 20 (32.3) | 14 (22.6) |
| Definitely agree | 20 (32.3) | 47 (75.8) |
| **PSYCH: confident** |  |  |
| Definitely disagree | 3 (4.8) | 0 |
| Somewhat disagree | 25 (40.3) | 1 (1.6) |
| Somewhat agree | 13 (21.0) | 11 (17.7) |
| Definitely agree | 21 (33.9) | 50 (80.6) |
| **PSYCH: attractive** |  |  |
| Definitely disagree | 5 (8.1) | 2 (3.2) |
| Somewhat disagree | 28 (45.2) | 5 (8.1) |
| Somewhat agree | 16 (25.8) | 16 (25.8) |
| Definitely agree | 13 (21.0) | 39 (62.9) |
| **PSYCH: great** |  |  |
| Definitely disagree | 6 (9.7) | 0 |
| Somewhat disagree | 28 (45.2) | 3 (4.8) |
| Somewhat agree | 12 (19.4) | 12 (19.4) |
| Definitely agree | 16 (25.8) | 47 (75.8) |
