# Supplementary material for: Brazil HARMONY: Improved Satisfaction With Facial Appearance and Psychosocial and Social Well-Being Following Combined Facial Aesthetic Treatment
Source: Aesthet Surg J Open Forum. 2026 Sep 7;8:ojag159. doi: 10.1093/asjof/ojag159 (PMC13603039; doi:10.1093/asjof/ojag159)
Supplement: Supplementary file 1 [file ojag159_supplementary_data.zip › ASJOF-26-0027_Brazil HARMONY_Supplementary Table 4.docx]

**Supplementary Table 4. FACE-Q Social Function Summary (n=62)**

|  | **Baseline, n (%)** | **Final Study Visit, n (%)** |
| --- | --- | --- |
| **SOCIAL: first impressions** |  |  |
| Definitely disagree | 2 (3.2) | 0 |
| Somewhat disagree | 12 (19.4) | 3 (4.8) |
| Somewhat agree | 39 (62.9) | 24 (38.7) |
| Definitely agree | 9 (14.5) | 35 (56.5) |
| **SOCIAL: new person** |  |  |
| Definitely disagree | 2 (3.2) | 0 |
| Somewhat disagree | 23 (37.1) | 6 (9.7) |
| Somewhat agree | 27 (43.5) | 18 (29.0) |
| Definitely agree | 10 (16.1) | 38 (61.3) |
| **SOCIAL: meet people** |  |  |
| Definitely disagree | 4 (6.5) | 0 |
| Somewhat disagree | 16 (25.8) | 5 (8.1) |
| Somewhat agree | 27 (43.5) | 11 (17.7) |
| Definitely agree | 15 (24.2) | 46 (74.2) |
| **SOCIAL: make friends** |  |  |
| Definitely disagree | 1 (1.6) | 1 (1.6) |
| Somewhat disagree | 11 (17.7) | 1 (1.6) |
| Somewhat agree | 28 (45.2) | 19 (30.6) |
| Definitely agree | 22 (35.5) | 41 (66.1) |
| **SOCIAL: group situations** |  |  |
| Definitely disagree | 4 (6.5) | 0 |
| Somewhat disagree | 20 (32.3) | 6 (9.7) |
| Somewhat agree | 15 (24.2) | 14 (22.6) |
| Definitely agree | 23 (37.1) | 42 (67.7) |
| **SOCIAL: social situations** |  |  |
| Definitely disagree | 4 (6.5) | 0 |
| Somewhat disagree | 17 (27.4) | 2 (3.2) |
| Somewhat agree | 16 (25.8) | 15 (24.2) |
| Definitely agree | 25 (40.3) | 45 (72.6) |
| **SOCIAL: around strangers** |  |  |
| Definitely disagree | 6 (9.7) | 0 |
| Somewhat disagree | 25 (40.3) | 7 (11.3) |
| Somewhat agree | 21 (33.9) | 24 (38.7) |
| Definitely agree | 10 (16.1) | 31 (50.0) |
| **SOCIAL: room of people** |  |  |
| Definitely disagree | 9 (14.5) | 1 (1.6) |
| Somewhat disagree | 24 (38.7) | 9 (14.5) |
| Somewhat agree | 18 (29.0) | 18 (29.0) |
| Definitely agree | 11 (17.7) | 34 (54.8) |
