# Supplementary material for: Brazil HARMONY: Improved Satisfaction With Facial Appearance and Psychosocial and Social Well-Being Following Combined Facial Aesthetic Treatment
Source: Aesthet Surg J Open Forum. 2026 Sep 7;8:ojag159. doi: 10.1093/asjof/ojag159 (PMC13603039; doi:10.1093/asjof/ojag159)
Supplement: Supplementary file 1 [file ojag159_supplementary_data.zip › ASJOF-26-0027_Brazil HARMONY_Supplementary Table 5.docx]

**Supplementary Table 5. FACE-Q Satisfaction with Skin Summary (n=62)**

|  | **Baseline, n (%)** | **Final Study Visit, n (%)** |
| --- | --- | --- |
| **SKIN: end of day** |  |  |
| Very dissatisfied | 24 (38.7) | 1 (1.6) |
| Somewhat dissatisfied | 31 (50.0) | 5 (8.1) |
| Somewhat satisfied | 6 (9.7) | 22 (35.5) |
| Very satisfied | 1 (1.6) | 34 (54.8) |
| **SKIN: healthy** |  |  |
| Very dissatisfied | 14 (22.6) | 1 (1.6) |
| Somewhat dissatisfied | 31 (50.0) | 4 (6.5) |
| Somewhat satisfied | 17 (27.4) | 16 (25.8) |
| Very satisfied | 0 | 41 (66.1) |
| **SKIN: attractive** |  |  |
| Very dissatisfied | 18 (29.0) | 1 (1.6) |
| Somewhat dissatisfied | 30 (48.4) | 5 (8.1) |
| Somewhat satisfied | 10 (16.1) | 22 (35.5) |
| Very satisfied | 4 (6.5) | 34 (54.8) |
| **SKIN: smooth** |  |  |
| Very dissatisfied | 14 (22.6) | 1 (1.6) |
| Somewhat dissatisfied | 22 (35.5) | 4 (6.5) |
| Somewhat satisfied | 25 (40.3) | 17 (27.4) |
| Very satisfied | 1 (1.6) | 40 (64.5) |
| **SKIN: clear** |  |  |
| Very dissatisfied | 26 (41.9) | 2 (3.2) |
| Somewhat dissatisfied | 21 (33.9) | 9 (14.5) |
| Somewhat satisfied | 10 (16.1) | 21 (33.9) |
| Very satisfied | 5 (8.1) | 30 (48.4) |
| **SKIN: refreshed** |  |  |
| Very dissatisfied | 27 (43.5) | 1 (1.6) |
| Somewhat dissatisfied | 25 (40.3) | 3 (4.8) |
| Somewhat satisfied | 8 (12.9) | 17 (27.4) |
| Very satisfied | 2 (3.2) | 41 (66.1) |
| **SKIN: hydrated** |  |  |
| Very dissatisfied | 19 (30.6) | 2 (3.2) |
| Somewhat dissatisfied | 25 (40.3) | 1 (1.6) |
| Somewhat satisfied | 18 (29.0) | 18 (29.0) |
| Very satisfied | 0 | 41 (66.1) |
| **SKIN: wake up** |  |  |
| Very dissatisfied | 25 (40.3) | 1 (1.6) |
| Somewhat dissatisfied | 25 (40.3) | 7 (11.3) |
| Somewhat satisfied | 10 (16.1) | 23 (37.1) |
| Very satisfied | 2 (3.2) | 31 (50.0) |
| **SKIN: radiant** |  |  |
| Very dissatisfied | 22 (35.5) | 1 (1.6) |
| Somewhat dissatisfied | 31 (50.0) | 4 (6.5) |
| Somewhat satisfied | 9 (14.5) | 20 (32.3) |
| Very satisfied | 0 | 37 (59.7) |
| **SKIN: tone** |  |  |
| Very dissatisfied | 0 | 0 |
| Somewhat dissatisfied | 24 (38.7) | 7 (11.3) |
| Somewhat satisfied | 23 (37.1) | 18 (29.0) |
| Very satisfied | 3 (4.8) | 37 (59.7) |
| **SKIN: pores** |  |  |
| Very dissatisfied | 21 (33.9) | 3 (4.8) |
| Somewhat dissatisfied | 26 (41.9) | 6 (9.7) |
| Somewhat satisfied | 14 (22.6) | 24 (38.7) |
| Very satisfied | 1 (1.6) | 29 (46.8) |
| **SKIN: even-colored** |  |  |
| Very dissatisfied | 24 (38.7) | 3 (4.8) |
| Somewhat dissatisfied | 24 (38.7) | 8 (12.9) |
| Somewhat satisfied | 13 (21.0) | 20 (32.3) |
| Very satisfied | 1 (1.6) | 31 (50.0) |
